# Uterine Adenomyosis Treated by Linzagolix, an Oral Gonadotropin-Releasing Hormone Receptor Antagonist: A Pilot Study with a New ’Hit Hard First and then Maintain’ Regimen of Administration

**DOI:** 10.3390/jcm10245794

**Published:** 2021-12-10

**Authors:** Jacques Donnez, Olivier Donnez, Jean Tourniaire, Michel Brethous, Elke Bestel, Elizabeth Garner, Sébastien Charpentier, Andrew Humberstone, Ernest Loumaye

**Affiliations:** 1 Medical School, Université Catholique de Louvain, 1200 Brussels, Belgium; 2 Société de Recherche pour l’infertilité (SRI), 143 Avenue Grandchamp, 1150 Brussels, Belgium; 3 Polyclinique Urbain V (ELSAN Group), Institut du Sein et de Chirurgie Gynécologique d’Avignon, 84000 Avignon, France; pr.olivier.donnez@gmail.com; 4 Clinique Rhône Durance (ELSAN Group), Department of Radiology, 84000 Avignon, France; jean.tourniaire@gmail.com; 5 ObsEva SA, 1228 Geneva, Switzerland; michel.brethous@obseva.ch (M.B.); elke.bestel@obseva.ch (E.B.); sebastien.charpentier@obseva.ch (S.C.); andrew.humberstone@obseva.ch (A.H.); ernest.loumaye@obseva.ch (E.L.); 6 ObsEva Inc., Boston, MA 02101, USA; elizabeth.garner@obseva.com

**Keywords:** adenomyosis, treatment, GnRH antagonist, linzagolix

## Abstract

(1) Background: The aim of the present pilot study was to study the effect of a new oral gonadotropin-releasing hormone antagonist on adenomyosis. (2) Methods: Eight premenopausal women, aged between 37 and 45 years, presenting with heavy menstrual bleeding, pelvic pain, and dysmenorrhea due to diffuse and disseminated uterine adenomyosis, confirmed by magnetic resonance imaging (MRI), received 200 mg linzagolix once daily for a period of 12 weeks, after which they were switched to 100 mg linzagolix once daily for another 12 weeks. The primary efficacy endpoint was the change in volume of the adenomyotic uterus from baseline to 24 weeks, evaluated by MRI. Secondary efficacy endpoints included the change in uterine volume from baseline to 12 and 36 weeks by MRI, and also weeks 12, 24, and 36 assessed by transvaginal ultrasound (TVUS). Other endpoints were overall pelvic pain, dysmenorrhea, non-menstrual pelvic pain, dyspareunia, amenorrhea, quality of life measures, bone mineral density (BMD), junctional zone thickness, and serum estradiol values. (3) Results: Median serum estradiol was suppressed below 20 pg/mL during the 12 weeks on linzagolix 200 mg, and maintained below 60 pg/mL during the second 12 weeks on linzagolix 100 mg. At baseline, the mean ± SD uterine volume was 333 ± 250 cm^3^. After 24 weeks of treatment, it was 204 ± 126 cm^3^, a reduction of 32% (*p* = 0.0057). After 12 weeks, the mean uterine volume was 159 ± 95 cm^3^, a reduction of 55% from baseline (*p* = 0.0001). A similar pattern was observed when uterine volume was assessed by TVUS. Improvements in overall pelvic pain, dysmenorrhea, non-menstrual pelvic pain, dyspareunia, and dyschezia, as well as quality of life measured using the EHP-30 were also observed. Mean percentage BMD loss at 24 weeks was, respectively, −2.4%, −1.3%, and −4.1% for the spine, femoral neck, and total hip. The most common adverse events were hot flushes, which occurred in 6/8 women during the first 12 weeks, and 1/8 women between 12 and 24 weeks. (4) Conclusions: Linzagolix at a dose of 200 mg/day reduced uterine volume, and improved clinically relevant symptoms. Treatment with 100 mg thereafter retains the therapeutic benefits of the starting dose while minimizing side effects. This ‘hit hard first and then maintain’ approach may be the optimal way to treat women with symptomatic adenomyosis.

## 1. Introduction

The current definition of adenomyosis is largely based on a publication by Bird et al. [1], who defined it as benign invasion of the endometrium into the myometrium, producing a diffusely enlarged uterus which microscopically exhibits ectopic, non-neoplastic, endometrial glands, and stroma surrounded by hypertrophic-hyperplastic musculature [1]. Currently, adenomyosis is described as an estrogen-dependent benign uterine disease, characterized by the presence of endometrial tissue inside the myometrium to a depth of at least 2.5 mm, often surrounded by hyperplastic and hypertrophic smooth muscle [2,3]. Adenomyosis is considered focal when a nodular collection is identified, or diffuse when glands and stroma are dispersed throughout the myometrium [4,5,6]. Typical symptoms of adenomyosis include chronic and intense pelvic pain, abnormal uterine bleeding, and infertility, although some patients are asymptomatic [7]. Improvements in imaging techniques, such as transvaginal ultrasonography (TVUS) and magnetic resonance imaging (MRI), have facilitated its diagnosis, leading to major advances in the field [4,5,6,7,8]. The estimated prevalence of adenomyosis among patients in reproductive age is 20–30% [9,10,11], and its association with deep endometriosis is not uncommon [4,5,6,7,8,9,10,11,12]. Increased numbers of diagnoses, including those in younger patients [13], combined with a growing trend to delay the first pregnancy, emphasize the importance of understanding the mechanisms responsible for its development, and proposing new medical therapies. Despite its prevalence and severity of symptoms, the pathogenesis of adenomyosis has not been elucidated, but there is strong evidence that estrogens play an important role in its development [2].

The principal objective of treating uterine adenomyosis is symptom management. Treatment options for women are limited and involve the use of analgesics or off-label hormone therapies. In a recent review, Cope et al. [14] stressed that there is very little specific information available about medical therapy. No drug is currently approved for the treatment of adenomyosis, but a number of hormones can be used off-label to alleviate symptoms [15]. Progestins, including dienogest and the levonorgestrel-releasing intrauterine system (LNG-IUS), show reasonable efficacy, but only if adenomyosis is limited and close to the uterine cavity [14,15,16]. In case of moderate or severe (full-thickness) disease, these drugs are not effective.

New medications, such as selective progesterone receptor modulators (SPRMs), are also ineffective, as SPRMs induce endometrial changes, known as progesterone receptor modulator-associated endometrial changes (PAECs), in intramyometrial endometrium [17,18]. Conway et al. reported worsening of several ultrasound characteristics of adenomyosis concomitant with an aggravation of symptoms in adenomyosis patients treated with ulipristal acetate (UPA) [18].

As adenomyosis is essentially estrogen-dependent, hormone therapies inhibiting ovulation, and reducing circulating estrogens may prevent cyclic intramyometrial growth of endometrial glands, based on the notion that the response of eutopic and ectopic endometrium to estrogens is typically similar [2]. In the view of Vannuccini and Petraglia [15,16], and Cope et al. [14], use of a gonadotropin-releasing hormone (GnRH) agonist for management of adenomyosis-related pain and bleeding should be considered as second-line therapy, and only for short-term administration because of its severe hypoestrogenic side effects, initial flare-up effect, and slow reversibility.

According to a recent report, oral GnRH antagonists may constitute a promising new potential treatment option, allowing dose-dependent control of estradiol (E2) levels [19,20]. In theory, it could reduce the occurrence of ectopic endometrial implants in the myometrium, relieve adenomyosis-associated pain, diminish uterine volume, and lower the prevalence of hypoestrogenic side effects by modulating the dosage [19,20].

The aim of the current pilot study was to investigate the safety and efficacy of linzagolix, a new orally active GnRH antagonist, in women with confirmed moderate or severe adenomyosis. It was an exploratory, open-label, single-arm pilot study evaluating the efficacy of a once-daily regimen of 200 mg linzagolix for 12 weeks, followed by a further 12 weeks on 100 mg. The aim was to first fully suppress serum E2 for 12 weeks, and then to provide maintenance for 12 weeks with partial suppression of serum E2.

## 2. Materials and Methods

### 2.1. Trial Design and Overview 

This was a pilot study conducted in a single center. It involved a screening period, during which adenomyosis symptoms and diagnostic criteria were evaluated over at least 4 weeks and included at least one menstruation, followed by a 24-week treatment period. Subjects completing the 24-week treatment period were followed-up for another 12 weeks. The trial was approved by the Ethics Committee and French National Agency for Drug and Health Product Safety, and performed in accordance with International Conference on Harmonization guidelines, all applicable regulations, and the ethical principles of the Declaration of Helsinki. The trial was registered on EudraCT: 2017-004-042-14. All potential study subjects provided written informed consent prior to any screening activities. The sponsor, ObsEva SA, designed the study, and analyzed the data with the lead authors (Donnez J and Donnez O), while a contract research organization, Atlanstat, managed the trial. The investigators, contract research organization, and study sponsor jointly conducted the trial, and gathered the data. All the authors had full access to these data. The first draft of the manuscript was written by a medical doctor (J.D.), and all the authors provided feedback on all versions of the manuscript. All the authors can attest to the completeness and accuracy of the data and analyses, and adherence to the trial protocol.

### 2.2. Patients

Premenopausal women aged 18 to 48 years with regular menstrual cycles and a confirmed MRI diagnosis of diffuse adenomyosis according to the classification of Bazot and Daraï [8] were included in the study. In addition, they had to have abnormal uterine bleeding, moderate to severe pain according to the mB&B classification for two symptoms (deep dyspareunia, pelvic pain, or dysmenorrhea), and a junctional zone width ≥12 mm. Subjects were excluded if they had liver enzyme anomalies, osteoporosis, or other metabolic bone disease. There was no bone mineral density (BMD) cut-off for participation. Women were also excluded if they had recently taken specific medications, such as oral contraceptives (1 month), GnRH analogues (3 to 6 months), or systemic glucocorticoids (1 month), with a specified wash-out period (in brackets) for each based on the time period these medications might be expected to have a continued effect. All trial subjects were instructed to use non-hormonal, double-barrier contraception, such as condoms or a diaphragm combined with a spermicide.

### 2.3. Trial Procedures and Assessments

Eligible subjects received 200 mg/day linzagolix for 12 weeks, after which they were switched to 100 mg linzagolix for 12 additional weeks of treatment. Patients were required to complete a daily paper diary to record uterine bleeding, dyschezia, dyspareunia, and medication intake, starting during the screening period, and continuing throughout the 24 weeks of treatment. Overall pain and serum E2 levels, and other assessments described below were assessed at monthly clinic visits. BMD of the lumbar spine, hip, and femoral neck were measured using dual-energy x-ray absorptiometry at baseline and 24 weeks; readings and blinded scans were interpreted centrally.

The primary efficacy endpoint was the change in volume of the adenomyotic uterus from baseline to week 24, evaluated by MRI. Secondary efficacy endpoints included the change in volume of the adenomyotic uterus from baseline to weeks 12 and 36 by MRI, and also weeks 12, 24, and 36 by TVUS. In our opinion, based on the work by Bazot and Daraï [8], MRI is a more accurate method to determine the junctional zone (JZ) thickness, as it is more difficult to find the limit between the JZ and normal myometrium by TVUS. Other secondary endpoints were the mean change from baseline to weeks 12, 24, and 36 in the thickest section of the anterior and posterior part of the uterine myometrium (sagittal assessment), and the largest diameter of the JZ of the observed adenomyosis assessed by MRI.

We also investigated changes from baseline to weeks 12, 24, and 36 in dysmenorrhea, non-menstrual pelvic pain, and dyspareunia according to the modified Biberoglu and Behrman (mB&B) scale, global pelvic pain over the preceding 4-week period using the numerical rating scale (NRS), the average monthly dyspareunia score defined as the mean of available daily dyspareunia scores over the preceding 4-week period evaluated by the verbal rating scale (VRS) and the NRS, the average monthly dyschezia score defined as the mean of weekly dyschezia scores over the preceding 4-week period using the NRS, and the comprehensive Endometriosis Health Profile (EHP-30) questionnaire. Time to amenorrhea was also considered a secondary endpoint, as was the absence of uterine bleeding (or spotting only) during the 4-week period prior to weeks 4, 8, 12, 16, 20, and 24. Assessments were made using a simplified bleeding scale. Patient global impression of change (PGIC) was assessed at weeks 0, 12, 24, and 36.

Safety evaluations included treatment-emergent adverse event (TEAE) frequency and severity (including hot flushes), endometrial assessments, and laboratory measurements (hematology, coagulation parameters, hemoglobin, biochemistry, E2, and lipids). Other safety criteria, namely weight, vital signs, electrocardiograms, and gynecological findings, were also recorded.

### 2.4. Statistical Analysis

Hypothesis testing was conducted based on change from baseline assessments for reduction in uterine volume by 24 weeks, testing the null hypothesis of no change from baseline versus the alternative hypothesis of a change (increase/decrease) from baseline. Two-sided hypothesis tests were performed at a nominal type I error rate (alpha) of 0.05. Calculated *p*-values were used primarily as a measure of evidence against the null hypothesis, rather than a formal statement of statistical significance.

The observed change from baseline was described using summary statistics (with appropriate transformations to normalize data as necessary). A formal statistical assessment of change was carried out at each timepoint using repeated-measures methods with two-sided 5% significance levels. The statistical model included weeks (categorical), baseline, and the interaction between weeks and baseline as covariates.

Safety and tolerability profiles were assessed versus baseline conditions, and descriptive statistics were produced where applicable. No formal hypothesis testing of safety data was undertaken. Treatment-emergent analysis was done for adverse events, which were coded according to the MedDRA preferred term and system organ class. Tabulations by severity and drug-relatedness were also drawn up.

## 3. Results

### 3.1. Patients

A total of 10 patients were screened, 8 of whom were included and completed 24 weeks of treatment. The women were aged between 37 and 45 years (median: 43 years). Their mean ± SD weight was 75 ± 19 kg. All patients reported heavy menstrual bleeding, pelvic pain, dysmenorrhea, and some degree of dyspareunia. The baseline mean ± SD composite pelvic pain and physical sign score using the modified mB&B was 10.1 ± 2.5, indicating severe symptoms.

### 3.2. Primary Efficacy Endpoint

**Figure 1:**
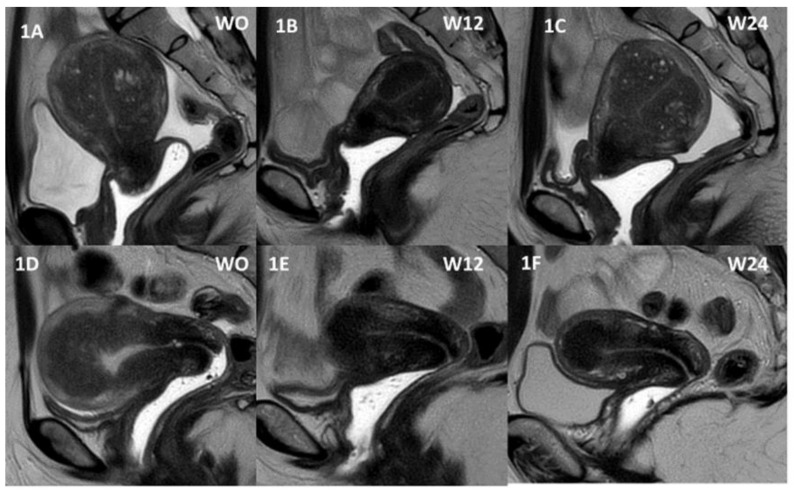
(**A**–**C**) Patient 1. (**A**) Magnetic resonance images showing an enlarged uterus with diffuse and disseminated adenomyosis at baseline. (**B**) After 12 weeks of GnRH antagonist therapy (daily dose 200 mg linzagolix), a significant reduction is observed in uterine size and adenomyotic lesions. (**C**) Patient was thereafter switched to linzagolix 100 mg/day, some regrowth was observed, but the volume remains significantly lower when compared to baseline. (**D**–**F**) Patient 2. MRI images at weeks 0, 12, and 24.

The primary endpoint was defined as change from baseline in uterine volume by 24 weeks measured by MRI (Figure 1).

**Figure 2:**
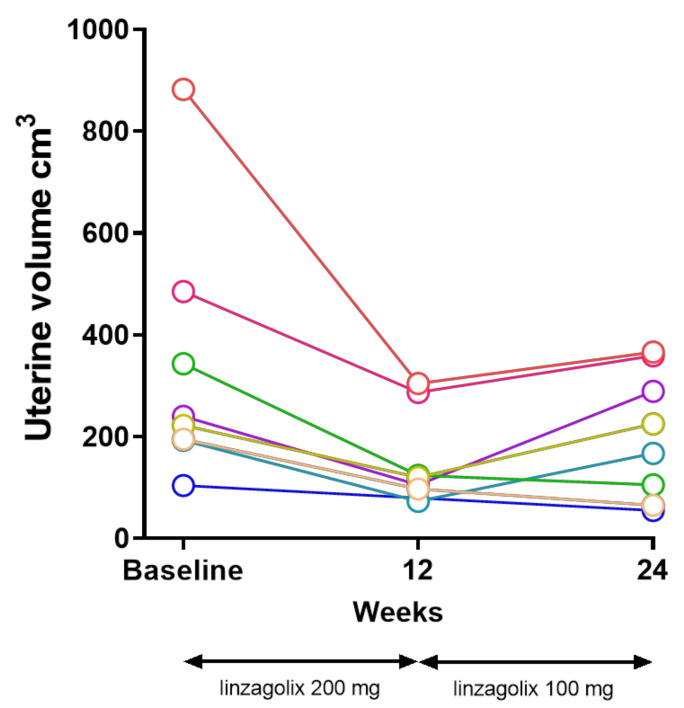
Uterine volume decreased significantly in all cases under linzagolix 200 mg/day. Some regrowth of the adenomyotic lesions was observed under partial E2 suppression with linzagolix 100 mg/day. In all cases but one, the volume at week 24 remained systematically less than at baseline.

The mean ± SD uterine volume was 333 ± 250 cm^3^ at baseline, and 204 ± 126 cm^3^ at 24 weeks. The estimated mean decrease from baseline at 24 weeks was 139 mL (95% CI 58, 220, *p* = 0.0057), corresponding to a mean 32% decrease (Figure 2 and Table 1).

### 3.3. Secondary Efficacy Endpoints

At 12 weeks, the mean ± SD uterine volume assessed by MRI was 159 ± 95 cm^3^. The estimated mean decrease from baseline was 198 mL (95% CI 156, 241, *p* < 0.0001), corresponding to a mean 55% decrease (Table 1).

When monitored by ultrasound, the mean ± SD uterine volume was 213 ± 171 cm^3^ at baseline, 88 ± 59 cm^3^ at 12 weeks, and 104 ± 61 at 24 weeks. The estimated mean decreases from baseline were 137 mL (95% CI 105, 169, *p* < 0.001) at 12 weeks, and 115 mL (95% CI 71, 159, *p* = 0.0007) at 24 weeks, corresponding to 58% and 39% decreases, respectively (Table 1). In five out of eight women, the uterine volume remained similar between 12 and 24 weeks, and in the other three women, there was some increase in uterine volume between 12 and 24 weeks, though remaining below baseline at 24 weeks.

The mean ± SD maximum values of the anterior and posterior wall measured by MRI were 36 ± 13 mm at baseline, 28 ± 10 mm at 12 weeks, and 31 ± 10 mm at 24 weeks, corresponding to mean 24% and 12% decreases from baseline, respectively. JZ thickness was 29 ± 12 mm at baseline, 19 ± 12 mm at 12 weeks, and 23 ± 11 mm at 24 weeks, corresponding to mean 38% and 20% decreases from baseline, respectively. The mean ± SD endometrial thickness was 10.6 ± 3.8 at baseline, and decreased to 4.1 ± 1.8 mm at 12 weeks, and 4.5 ± 4.4 mm at 24 weeks. An endometrial thickness of >5 mm was observed in only one woman with an E2 level of 238 pg/mL.

Mean global pelvic pain, dyspareunia, and dyschezia scores, self-reported by the women in their diary on a numerical rating scale of 0 to 10, decreased over time. The mean ± SD global pelvic pain scores were 8.4 ± 1.1 at baseline, 5.0 ± 3.3 at 4 weeks (*p* = 0.034), 2.4 ± 2.4 at 8 weeks (*p* = 0.0005), 2.4 ± 3.4 at 12 weeks (*p* = 0.0035), and 0.6 ± 0.7 at 24 weeks (<0.0001). The mean ± SD dyspareunia scores were 1.2 ± 1.2 at baseline, 1.0 ± 1.0 at 12 weeks (*p* = 0.155), and 0.1 ± 0.2 at 24 weeks (*p* < 0.001). The mean ± SD dyschezia scores were 2.7 ± 2.8 at baseline, to 1.9 ± 2.1 at 12 weeks (*p* = 0.212), and 1.6 ± 2.2 at 24 weeks (*p* = 0.101).

Similar decreases were observed in dysmenorrhea, dyspareunia, non-menstrual pelvic pain, total pelvic pain, and pelvic tenderness upon examination using the mB&B severity scale (Table 2).

Improvement in quality of life was demonstrated by marked reduction in all EHP-30 domain scores (pain, control, powerlessness, emotional well-being, social support, and self-image) at 12 and 24 weeks (Table 3).

Seven of the eight women (87.5%) recorded ‘very much’ improved on the PGIC scale by week 12, and eight out of eight reported ‘much’ or ‘very much’ improved by week 24.

Amenorrhea, defined as no uterine bleeding reported for at least 35 days, was reported for six out of six women at 12 weeks, and four out of seven women at 24 weeks (data were missing for the other women). Mean time from treatment onset to start of amenorrhea was 21.3 ± 13.4 days (n = 6, median: 22 days, range 4–43 days).

### 3.4. Estradiol Levels

**Figure 3:**
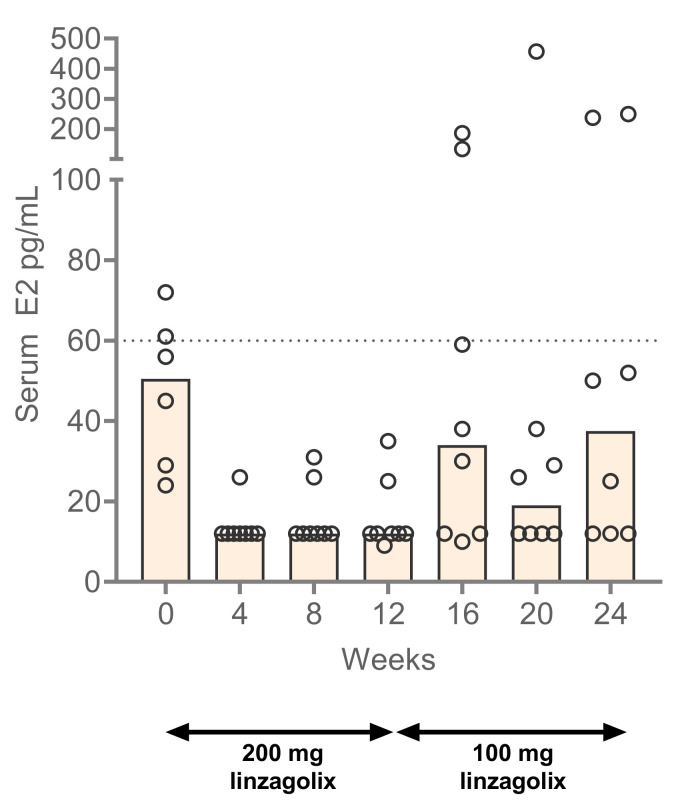
Serum estradiol (E2) levels at baseline and under linzagolix 200 mg and 100 mg. Shaded boxes indicate median serum E2 levels, and open circles indicate individual values.

Serum E2 was fully suppressed during the first 12 weeks; median serum E2 was 12 pg/mL by week 4, which was maintained up to week 12. After patients were switched to 100 mg/day linzagolix, median serum E2 values increased to 34, 19, and 37.5 pg/mL at weeks 16, 20, and 24, respectively (Figure 3). Two patients experienced high E2 levels under linzagolix 100 mg, each patient twice. Linzagolix induced a partial suppression of E2. The goal was to reach the target area of E2 between 20 and 50 pg/mL, but some women may escape the therapy. Among the eight women, all were in amenorrhea under linzagolix 200 mg/day, and four of them remained in amenorrhea under linzagolix 100 mg/day.

### 3.5. Bone Mineral Density

**Figure 4:**
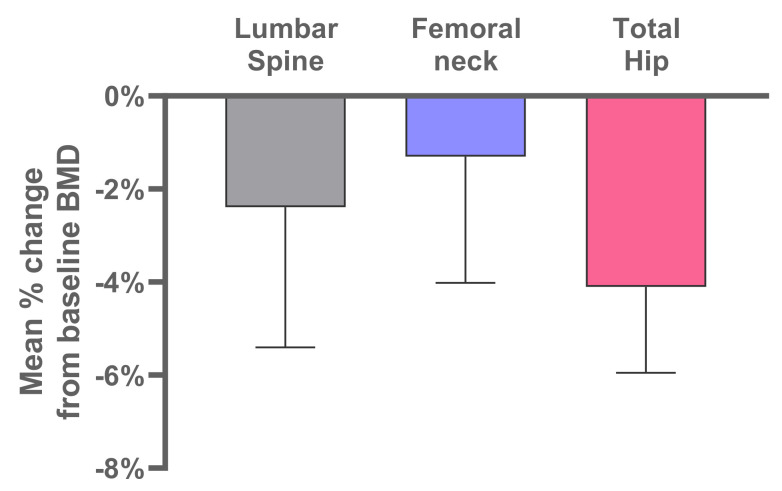
Bone mineral density percentage change from baseline (CFB) at week 24. Error bars show 95% CI.

**Figure 5:**
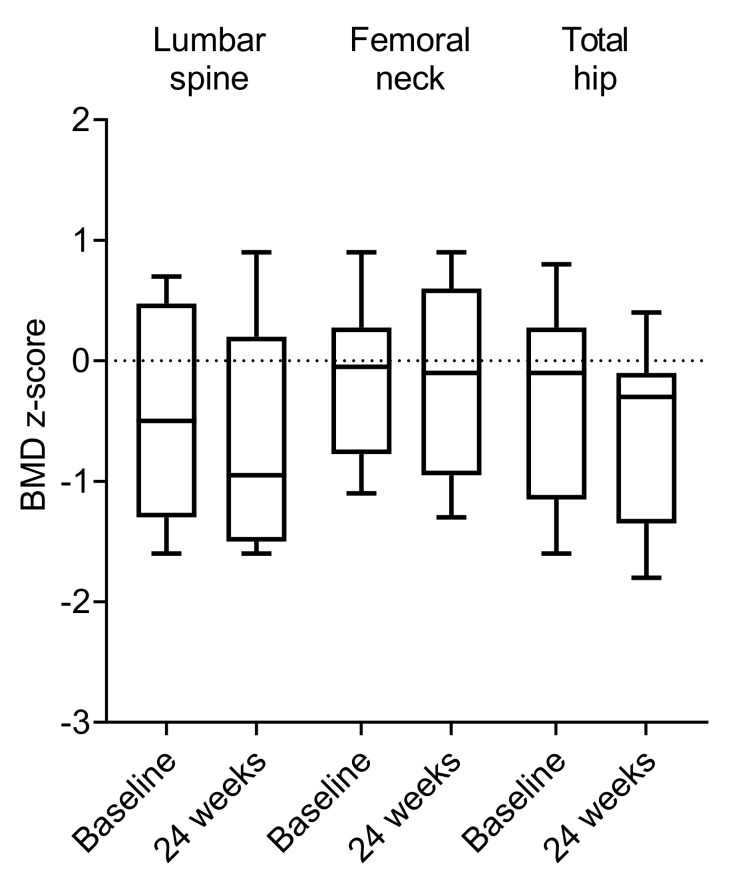
Bone mineral density Z-scores at baseline and week 24. Boxes show medians, Q1, and Q3, and whiskers show range.

Mean ± SD percentage decreases from baseline in BMD at 24 weeks were −2.39 ± 3.60% in the lumbar spine, −1.30 ± 3.25% in the femoral neck, and −4.10 ± 2.20% in the total hip (Figure 4). Median Z-scores and total ranges for the lumbar spine, femoral neck, and total hip at baseline and week 24 did not change markedly over time (Figure 5).

### 3.6. Treatment-Emergent Adverse Events

The most frequently reported TEAEs related to the study drug were mild and moderate hot flushes, which were reported in six out of eight women up to 12 weeks, and in one woman from 12 to 24 weeks. Also related to the study drug, decline or loss of libido was reported in three out of eight women. The most frequently TEAEs reported as not related to the study drug were fatigue in three women, and anxiety in two women. No deaths occurred during the trial.

### 3.7. Clinical Laboratory Tests

No increase was noted in either ALT or AST. One woman with a gamma GT level of 63 IU/l at baseline exhibited levels of 94, 151, 159, and 118 IU/l at weeks 4, 8, 12, and 24, respectively, without any concomitant rise in total bilirubin. Mean serum high-density lipoprotein (HDL) cholesterol, low-density lipoprotein (LDL) cholesterol, cholesterol, and triglyceride levels were similar at baseline, week 12, and week 24. No meaningful changes were observed between baseline, week 12, and 24 in terms of biochemical analyses, total protein, urea, creatinine, or uric acid values.

## 4. Discussion

Despite its prevalence and severity of symptoms, the pathogenesis of adenomyosis has not yet been elucidated, and symptomatic medical treatment remains challenging. No single theory can explain all the different phenotypes of the disease, but three main hypotheses have been proposed [2]. The first and foremost involves invagination of the endometrial basalis, hyperestrogenism, hyperperistalsis, and tissue injury and repair (TIAR) activation [21,22]. According to two further alternative theories, adenomyotic lesions may originate from metaplasia of displaced embryonic pluripotent Müllerian remnants, or differentiation of adult stem cells that are present in the myometrium [2]. Whatever the theory, there is strong evidence that estrogens play an important role in the development of uterine adenomyosis and the resulting symptoms [2].

The principal objective of treating adenomyosis is symptom management. Treatment requires a long-term management plan, as the disease often has chronic negative impact on QoL until menopause is reached [16]. There are limited data on medical therapy for adenomyosis. Progestogens and combined oral contraceptives are not very effective, as progesterone resistance in adenomyotic endometrium and stroma is considered another characteristic of uterine adenomyosis, similar to observations in deep endometriotic nodules that are frequently associated with uterine adenomyosis [4,5,12,23,24]. Decreased expression of progesterone receptor B in adenomyotic lesions has been reported to be the underlying cause of progesterone resistance, and is thought to be epigenetically regulated [25,26].

In a recent review, Cope et al. [14] stressed that there is very little specific information available on medical therapy, and that further high-quality studies are much needed. No drug is currently approved for the treatment of adenomyosis, but a number of hormones are often used off-label to alleviate symptoms. The LNG-IUS did initially look promising for first-line therapy, but its use should be limited to mild and moderate cases of adenomyosis without sizable increases in uterine volume. GnRH agonists are effective at treating adenomyosis symptoms, but they have numerous limitations, including a delayed therapeutic impact because of the flare-up effect, excessive suppression of E2 to less than 20 pg/mL (with related unfavorable side effects), inability to titrate E2 levels, and unpredictable reversibility of treatment when injectable depot forms of GnRH agonists are used [15,16,20].

There is clearly a large unmet need for improved long term medical therapies for adenomyosis [18]. According to the estrogen threshold hypothesis suggested by Barbieri [26], managing estrogen levels to minimize withdrawal symptoms and sequelae, while maintaining efficacy in terms of mitigation of symptoms, may constitute a viable treatment option [27]. Oral GnRH antagonists have emerged as a potential alternative to allow dose-dependent control of E2 levels [20]. In addition to their unique capacity to modulate E2 suppression, another advantage of orally active GnRH antagonists over GnRH agonist depot formulations is the absence of the flare-up effect, hence avoiding initially worsening symptoms, and rapid reversibility [20]. Several studies have recently demonstrated that oral, non-peptide GnRH antagonists are effective at treating endometriosis-associated pain [27,28,29,30]. Since all pathways involved in the pathogenesis of adenomyosis also feature estrogens in a major role [2,3,4,5,6,7,8,9,10,11,12,13,14,15,16,17,18,19,20,21,22,23], the same therapy could be applied to symptomatic adenomyosis [31]. Indeed, the first case report describing a significant reduction in adenomyotic lesions with linzagolix was recently published [20].

In the present pilot study, it was decided to start with a once-daily dose of 200 linzagolix for 12 weeks to obtain rapid and full suppression of E2 levels, with the goal of inducing a strong initial effect on the adenomyotic lesions. This was followed by 12 weeks of therapy with once-daily 100 mg linzagolix, providing partial suppression of E2 with the aim to maintain efficacy, while decreasing side effects.

The desired impact of this dosage regimen of linzagolix on serum E2 levels was confirmed in this study. During the first 12 weeks of treatment with 200 mg linzagolix, median serum E2 levels were suppressed below 20 pg/mL, and when patients were switched to 100 mg/day linzagolix, median serum E2 levels rose to between 20 and 40 pg/mL, with occasional increases above 60 pg/mL in some individuals.

There were corresponding clinically and statistically significant decreases in uterine volume from baseline, corresponding to a mean 55% decrease at 12 weeks, and 32% at 24 weeks, demonstrating a marked decrease during the first 12 weeks of full E2 suppression, with some increase of mean uterine volume during the second 12 weeks of partial suppression with 100 mg linzagolix. A similar pattern was observed for other secondary endpoints, such as JZ, anterior and posterior wall and endometrial thickness, and the incidence of amenorrhea.

For other secondary pain endpoints, including overall pelvic pain, dysmenorrhea, non-menstrual pelvic pain, dyspareunia, and dyschezia, as well as the pain, control, powerlessness, emotional well-being, social support, and self-image scores for the EHP-30, improvements observed after 12 weeks were maintained or improved further by 24 weeks. Likewise, seven out of eight women reported ‘very much’ improved on the PGIC scale by week 12, and this improvement was fully maintained when the patients were switched to 100 mg, all of them reporting ‘much ‘or ‘very much’ improved.

When overall pelvic pain was assessed every 4 weeks using the patient diary, a significant decrease was noted by week 4, with further improvement and a stronger response by week 12, which was then maintained until week 24.

Mean percentage decrease in BMD in the lumbar spine, femoral neck, and total hip were, respectively, −2.4%, −1.3%, and −4.1% at 24 weeks. However, the BMD Z-scores remained in a similar range at 24 weeks as at baseline, suggesting that these % BMD losses are not clinically relevant. It should be noted that calcium and vitamin D supplementation were not provided in the trial protocol. Some of the BMD decrease observed in the study could be avoided with regular calcium and vitamin D administration in future studies and/or clinical use.

The incidence of hot flushes was higher during the first 12-week period (six out of eight women) compared to only one woman during the second 12-week period. Similar dose-related differences were observed in a previous study [29]. There were no changes in HDL cholesterol, LDL cholesterol, or cholesterol and triglyceride levels, which remained in the normal range from baseline to week 24. No subjects showed an increase above twice the upper limit of the normal range for ALT, AST, or bilirubin with respective doses of 200 mg and 100 mg linzagolix. No deaths were reported in the study. The women were all instructed to use dual non-hormonal contraception, and there were no pregnancies.

In conclusion, the results of this study provide evidence that linzagolix administered at a high dose for 12 weeks followed by a lower maintenance dose for a further 12 weeks to women with severe symptomatic adenomyosis substantially reduced uterine volume, decreased uterine bleeding, reduced pain symptoms, and improved quality of life at 12 and 24 weeks.

This is consistent with the mechanism of action of linzagolix, which leads to a rapid decline in serum E2 levels by competitively inhibiting GnRH receptors in the pituitary, avoiding the initial phase of stimulation (flare-up) observed with a GnRH agonist. Another advantage compared to a GnRH agonist is that E2 suppression can be modulated by changing doses (e.g., switching from 200 to 100 mg in the present study) to reduce hypoestrogenic side-effects, while efficacy is maintained in terms of control of bleeding, pain, and QoL.

We tested once-daily 200 mg linzagolix for 12 weeks, to exert an immediate effect with maximum efficacy, subsequently followed by 100 mg, to preserve the therapeutic benefits while minimizing side effects [19,20,31]. Based on these pilot data, the proposed new regimen of administration for linzagolix of hitting hard first to promptly obtain optimal potency, followed by a lower maintenance dose, warrants further assessment.

## Figures and Tables

**Table 1:** Uterine volume, anterior or posterior wall, junctional zone, and endometrial thickness.

	Baseline	Week 12	Week 24
n	8	7	8
Uterine Volume (cm^3^) by MRI			
Mean (SD)	333.0 (249.8)	158.6 (95.1)	203.9 (125.7)
Median (range)	231 (104, 882)	120 (73, 304)	196 (55, 366)
Mean change (SD)		−207.1 (170.1)	−129.1 (180.4)
Mean % change (SD)		−55.0 (9.6)	−32.4 (33.3)
Estimated mean change (95% CI)		−198.3 (−240.9, −155.6)	−138.8 (−219.7, −58.0)
p Value		<0.0001	0.0057
Uterine volume (cm^3^) by TVUS			
Mean (SD)	213.3 (171.3)	88.30 (59.30)	103.89 (61.18)
Median (range)	131 (102, 581)	57 (45, 186)	114 (29, 180)
Mean change (SD)		−140.2 (131.5)	−109.5 (141)
Mean % change (SD)		−57.70 (14.87)	−39.04 (45.16)
Estimated mean change (95% CI)		−137.1 (−168.8, −105.3)	−114.9 (−159.3, −70.6)
p Value		<0.0001	0.0007
Maximum value of anterior or posterior wall (mm) by MRI			
Mean (SD)	35.5 (13.0)	28.0 (10.2)	30.8 (10.2)
Median (range)	32 (25, 65)	23 (21, 48)	30.5 (18, 49)
Mean change (SD)		−9.0 (5.4)	−4.8 (7.4)
Mean % change (SD)		−23.9 (10.8)	−11.6 (18.2)
Maximum value of anterior or posterior wall (mm) by TVUS			
Mean (SD)	38.4 (13.1)	28.3 (8.3)	31.0 (10.5)
Median (range)	32.5 (30, 68)	24 (21, 42)	31.5 (20, 50)
Mean change (SD)		−11.1 (7.3)	−7.4 (7.9)
Mean % change (SD)		−27.0 (10.2)	−17.9 (21.1)
Junctional Zone (mm) by MRI			
Mean (SD)	29.0 (11.9)	19.3 (11.7)	23.3 (11.1)
Median (range)	25 (19, 57)	16 (8, 42)	24.5 (7, 44)
Mean change (SD)		−10.3 (3.1)	−5.8 (6.6)
Mean % change (SD)		−38.1 (14.7)	−20.2 (26.0)
Junctional Zone (mm) by TVUS			
Mean (SD)	24.5 (11.2)	11.7 (5.6)	14.9 (9.1)
Median (range)	22 (13, 49)	10 (4, 20)	14.5 (3, 28)
Mean change (SD)		−13.4 (7.5)	−9.6 (6.5)
Mean % change (SD)		−53.3 (11.6)	−41.4 (24.2)
Endometrial thickness (mm) by TVUS			
Mean (SD)	10.6 (3.8)	4.1 (1.8)	4.5 (4.4)
Median (range)	11.5 (4, 17)	4.0 (1, 6)	3.0 (2, 15)

**Table 2:** Individual and composite scores on the modified Biberoglu and Behrman symptom severity scale *.

		Baseline	Week 12	Week 24
Dysmenorrhea	n	8	8	8
	Mean (SD)	2.8 (0.5)	0.0 (0.0)	0.1 (0.4)
	Mean Change (SD)		−2.8 (0.5)	−2.6 (0.7)
Deep Dyspareunia	n	8	8	7
	Mean (SD)	2.1 (1.1)	0.6 (0.7)	0.0 (0.0)
	Mean Change (SD)		−1.5 (1.3)	−2.0 (1.2)
Non-Menstrual Pelvic Pain	n	8	8	8
	Mean (SD)	2.0 (0.8)	0.8 (1.0)	0.3 (0.5)
	Mean Change (SD)		−1.3 (1.3)	−1.8 (0.9)
Total Pelvic Pain Score	n	8	8	7
	Mean (SD)	6.9 (1.7)	1.4 (1.5)	0.3 (0.5)
	Mean Change (SD)		−5.5 (2.2)	−6.3 (1.8)
Pelvic Tenderness	n	8	7	8
	Mean (SD)	2.4 (0.7)	0.3 (0.5)	0.3 (0.5)
	Mean Change (SD)		−2.0 (0.6)	−2.1 (1.0)
Induration	n	8	7	8
	Mean (SD)	0.9 (0.6)	0.1 (0.4)	0.0 (0.0)
	Mean Change (SD)		−0.7 (0.8)	−0.9 (0.6)
Total Physical Sign Score	n	8	7	8
	Mean (SD)	3.3 (0.9)	0.4 (0.5)	0.3 (0.5)
	Mean Change (SD)		−2.7 (1.0)	−3.0 (1.1)
Composite Pelvic Pain Physical Sign Score	n	8	7	7
	Mean (SD)	10.1 (2.5)	1.7 (1.8)	0.6 (0.8)
	Mean Change (SD)		−8.4 (3.0)	−9.1 (2.7)

* based on Biberoglu, KO; Behrman, SJ. Dosage aspects of danazol therapy in endometriosis: short-term and long-term effectiveness. Am. J. Obstet. Gynecol. 1981;139:645.

**Table 3:** EHP-30 domain scores.

		Baseline	Week 12	Week 24
Pain score	n	8	8	8
	Mean (SD)	64.5 (21.1)	6.3 (17.7)	3.4 (6.4)
	Mean Change (SD)		−58.2 (23.3)	−61.1 (19.9)
Control and powerlessness score	n	8	8	8
	Mean (SD)	82.8 (14.3)	9.9 (23.4)	10.4 (16.4)
	Mean Change (SD)		−72.9 (21.0)	−72.4 (17.9)
Emotional well-being score	n	8	8	8
	Mean (SD)	60.4 (23.5)	9.4 (15.7)	8.3 (15.9)
	Mean Change (SD)		−51.0 (20.9)	−52.1 (21.2)
Social support score	n	8	8	8
	Mean (SD)	69.5 (31.9)	10.2 (24.1)	6.3 (17.7)
	Mean Change (SD)		−59.4 (30.3)	−63.3 (30.0)
Self-image score	n	8	8	8
	Mean (SD)	68.8 (34.7)	24.0 (36.6)	12.5 (20.9)
	Mean Change (SD)		−44.8 (44.1)	−56.3 (33.9)
